# Translation into Spanish, Transcultural Adaptation and Validation of the Foot Function Index Revised Short Form (FFI_RSF) Questionnaire

**DOI:** 10.3390/jcm14113638

**Published:** 2025-05-22

**Authors:** Pablo Cervera-Garvi, Mercedes Ortiz-Romero, Luis María Gordillo-Fernandez, Irene Garcia-Paya, Ana Belen Ortega-Avila, Salvador Diaz-Miguel

**Affiliations:** 1 Department of Nursing and Podiatry, Faculty of Health Sciences, University of Málaga, 29071 Málaga, Spain; pcervera@uma.es (P.C.-G.); irenegpaya@uma.es (I.G.-P.); salvadordiazm@uma.es (S.D.-M.); 2 Department of Podiatry, Faculty of Nursing, Physiotherapy and Podiatry, University of Seville, 41004 Seville, Spain; mortiz17@us.es (M.O.-R.); lgordillo@us.es (L.M.G.-F.); 3 Biomedical Research Institute (IbiS), 41013 Seville, Spain; 4 Biomedical Research Institute (IBIMA), 29590 Málaga, Spain

**Keywords:** foot, ankle, function, transcultural, validity, questionnaire properties

## Abstract

**Background/Objectives**: This study aimed to translate the Foot Function Index Revised Short Form (FFI RSF-Sp) into Spanish, perform a transcultural adaptation, and validate the resulting questionnaire on foot and ankle function. The revised instrument, derived from the original version of the FFI, incorporates additional subscales on ankle stiffness and overall quality of life that help produce a more comprehensive assessment and overcome some limitations. **Methods**: The final sample consisted of 306 participants from Malaga and Seville (Spain), all aged over 18 years and native Spanish speakers. These participants also completed the FAAM-Sp and VASFA-Sp validated questionnaires. The measurement properties of FFI RSF-Sp were evaluated in accordance with the Consensus-Based Standards for the Selection of Health Status Measurements Instruments (COSMIN) recommendations. **Results**: Factor analysis confirmed the existence of a five-factor structure, explaining 67.6% of the total variance, with an RMSEA of 0.0785 and a Tucker–Lewis index of 0.874. Internal consistency was excellent, with an overall Cronbach’s alpha of 0.963 and subscale values ranging from 0.776 to 0.886. Furthermore, moderate to strong correlations were obtained with the VASFA-Sp (r = −0.651) and FAAM ADL (r = −0.737, *p* < 0.001), and the obtained area under the curve value was 0.806 (*p* < 0.001), confirming the discriminatory capacity of the instrument. **Conclusions**: The FFI RSF-Sp is a valid, reliable, and sensitive instrument for assessing foot and ankle function in a Spanish-speaking population. The incorporation of additional dimensions improves the understanding of dysfunction, and the robust psychometric indicators of this questionnaire make it appropriate for use in clinical and research settings, facilitating the detection of functional alterations and enhancing treatment follow up.

## 1. Introduction

Many adults experience alterations and pathologies in the foot and/or ankle, which can severely impact on the correct function of these structures [1]. Various factors may be relevant to this condition, including gender (the prevalence of foot and ankle conditions is greater among women), body mass index (the prevalence is higher among people with obesity), and age (with a higher prevalence among adults) [2,3].

Patient Report Outcome Measure (PROM) questionnaires have long been used to obtain information about these alterations and monitor the outcomes of treatment, providing a perception of health problems from the patient’s own experience [4]. Several PROMs have been developed to identify problems related to foot health and function. One of the most widely used both in clinical practice and in research into foot and ankle disorders is the Foot Function Index (FFI) [5]. The FFI was developed by Budiman-Mak, Conrad, and Roach in 1991 [6] and has been in use for over 30 years. It consists of 23 questions grouped into the subcategories of pain, disability, and functional limitation, and has good psychometric properties [7]. Furthermore, it has been translated and adapted into many languages [8].

However, the original FFI has been criticised for not addressing psychosocial questions and aspects of the patient’s quality of life that may affect foot health, shortcomings that limit its application [9]. To this end, a revised version, the Foot Function Index Revised (FFI-R), was created in 2006, incorporating a new subcategory related to quality of life, together with pain, stiffness, disability, and functional limitation [10]. The addition of this new subscale, quality of life, provides the FFI-RSF with a more holistic perspective on foot function compared to its original version, since emotional state can influence an individual’s perception of their body, in this case, the foot.

The FFI-R was produced in two versions, the Foot Function Index Revised Long Form (FFI RLF) with 68 questions and the Foot Function Index Revised Short Form (FFI RSF) with 34 questions (spanning all five sub-categories). Like the original version, these revisions present solid psychometric properties [7].

To date, the only available versions of the FFI RSF are the original English instrument [10] and adaptations to Polish [11] and Norwegian [12]. There exists a Spanish adaptation of the original FFI [13], but not of the revised version with the additional subscales. The FFI-RSF can be used in a wider range of pathologies where pain or general quality of life influences patient function, allowing the use of a single tool. The objective of the present study is to translate and adapt the FFI RSF questionnaire into Spanish and analyse the psychometric properties of the resulting instrument by administering it to Spanish-speaking patients.

## 2. Materials and Methods

This study was approved by the corresponding Ethics Committee and was carried out in accordance with the Declaration of Helsinki. All participants were informed of the nature of the study and signed the consent form. The collection and custody of data were carried out based on current legislation following the guidelines of the Spanish Organic Law on the Protection of Personal Data and the General Data Protection Regulation of the European Union.

The measurement properties of FFI RSF-Sp were evaluated in accordance with the Consensus-Based Standards for the Selection of Health Status Measurements Instruments (COSMIN) recommendations [14].

Prior to data collection, a bibliographic search was carried out between October and December 2024 to determine how many cross-cultural adaptations had been made of the FFI-RSF questionnaire. This search showed that the Polish [11] and Norwegian [12] versions were the only prior adaptations, while the long-form original version of the FFI had been adapted and validated for many languages [8], with good reliability in assessing ankle and foot function.

### 2.1. Participants

Data for the study sample were collected between March and July 2024. The 321 participants were inhabitants of Malaga, at the Podiatry Teaching and Assistance Unit of the University of Málaga and at the Doctores del Pie Podiatry Clinic, Seville (Spain), and all met the following inclusion criteria: age over 18 years, native language Spanish, any level of education/professional training, in good health, and able to complete the questionnaire without assistance. Individuals included in the sample were asked whether they wished to participate voluntarily when attending Podiatry Centers for their treatments. If they agreed, the full questionnaire was then administered. For those questionnaires deemed invalid, their data were excluded and not considered in the final analysis.

### 2.2. Translation and Cross-Cultural Adaptation

**Figure 1:**
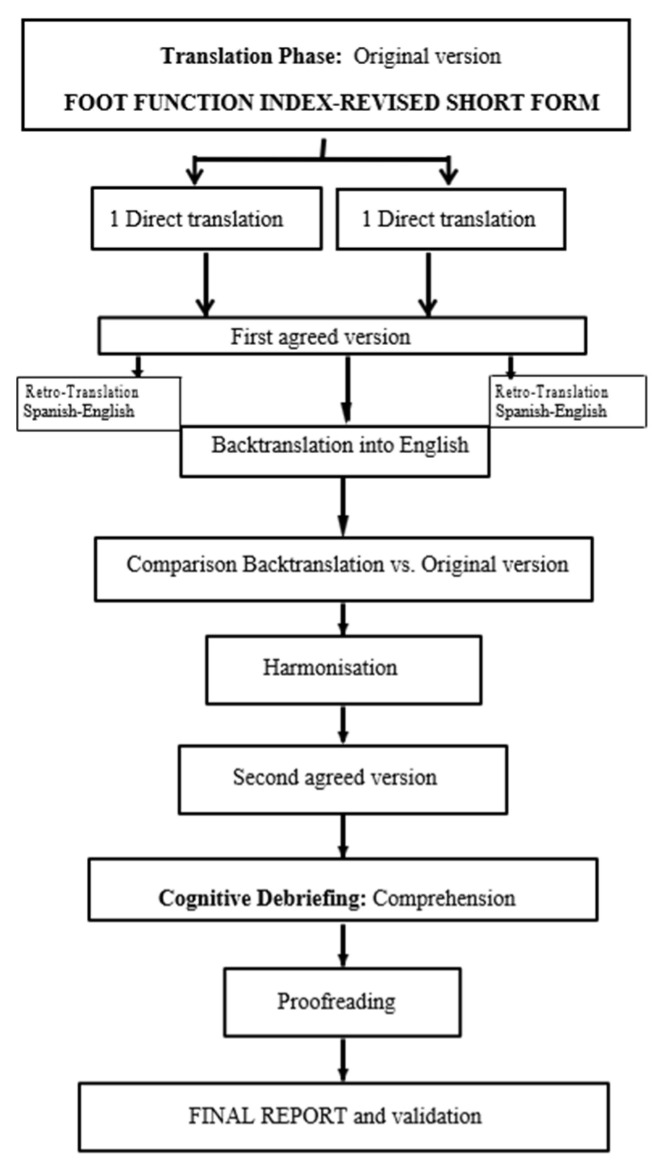
Cross-cultural adaptation process of the FFI-RSF-Sp.

The FFI RSF questionnaire was translated into Spanish in accordance with the guidelines of the Patient-Reported Outcomes Measurement Information System (PROMIS) and the International Society for Pharmacoeconomics and Outcomes Research (ISPOR) [15]. The protocol applied consisted of eight phases: (1) direct translation of the questionnaire into Spanish by two Spanish translators with expertise in health sciences. (2) By consensus, these individual translations were combined into a single version. (3) This single version was backtranslated into English by two bilingual native English speakers, blinded to the original version. (4) The study leader reviewed the back-translation for discrepancies. (5) A committee of experts, including patients, podiatrists, and translators, verified both translations, and difficult-to-understand items were rewritten for the final version. (6) The same committee verified cultural equivalence and developed a final version for pre-testing. (7) This was used in pre-trial studies with 20 patients to determine whether any changes were needed. (8) Finally, the study leader checked the translated versions for errors and typos and made any necessary corrections. Figure 1 presents a flowchart of the cross-cultural adaptation process.

### 2.3. Data Collection

All participants were first informed about the nature of the study and signed the corresponding consent forms. Before completing the questionnaires, each participant was interviewed and asked about their age, gender, height, weight, occupation, education background, sports activity, and any presence of diabetes, osteoarthritis, and/or diagnosed podiatric conditions. All then completed the Foot and Ankle Ability Measures scale (FAAM), the Visual Analogue Scale for the Foot and Ankle (VASFA), and the Foot Function Index Revised Short Form (FFI RSF). Finally, the participants were asked if they had had any difficulty in understanding the questions, and all responded that they had had no such problem; if there was a lack of data in the interview or in the questionnaires, they would be eliminated from the list of participants.

The FAAM-Sp is a self-administered questionnaire, cross-culturally adapted from the FAAM and validated for use in a Spanish-speaking population [16], which assesses foot and ankle functions related to the activities of daily living (ADL). The ADL scale consists of 15 items, scored on a five-point Likert system, ranging from 4 points (no difficulty) to 0 points (unable to perform the action). The score recorded is divided by the maximum potential score of the scale and multiplied by 100 to obtain a percentage, with a higher score meaning a higher level of function.

VASFA-Sp is a reliable, valid, and sensitive questionnaire that is suitable for measuring perceived foot and ankle function impairment in a Spanish-speaking population [17]. VASFA has 20 items grouped into 3 subscales: function (items 8–17 and 19), pain (items 2–5), and other complaints (items 1, 6, 7, 18, and 20). This instrument consists of 20 questions scored on a visual analogue scale ranging from 0 to 10 points, in which 0 is the worst function score and 10 is the optimal state score. A higher score represents better health status.

The FFI RSF is a 34-item questionnaire, derived from the 68-item FFI RF. For the total foot function assessment, the 34-item FFI-RSF is practically as good as the 68-item FFI-R [10]. The 34-item FFI RSF is further divided into five sub-categories: Pain (7 items), Stiffness (7 items), Disability (11 items), Functional Limitation (3 items), and Quality of Life (6 items). Each item is rated on a scale of 1 to 4, ranging from 1 (no difficulty) to 4 (severe difficulty). These scores are then totalled, divided by the maximum possible score, and multiplied by 100. Possible scores range from 0 to 100, with a lower score indicating better health.

### 2.4. Statistical Analysis

All statistical analyses were performed using SPSS v.25.0 and SPSS Amos v.26 statistical software (IBM Corporation, Armonk, NY, USA).

The internal structure of the questionnaire was evaluated through exploratory factor analysis, which confirmed the factors defined in the original version. A confirmatory factor analysis was then performed to determine the fit [18]. The cutoff score for FFI-RSF-Sp was assessed according to the receiver operating characteristic (ROC) curve and the area under the curve (AUC) [19]. Internal consistency was measured using Cronbach’s alpha, with values up to 0.7 considered fair, 0.8 to 0.9 good, and greater than or equal to 0.9 excellent [20]. Criterion validity was assessed by determining the Pearson correlations between the Spanish version of the FFI-RSF questionnaire and the FAAM-Sp and VASFA-Sp questionnaires. Results greater than or equal to 0.70 were considered to represent strong correlations, less than 0.70 moderate, and less than 0.30 poor [21]. Reliability was assessed by test–retest, administering the FFI-RSF-Sp questionnaire twice to 20 participants with a time interval of 7 days, and evaluated using intraclass correlation coefficients (ICC) [1,2]. An ICC value > 0.7 was considered excellent, 0.60–0.69 good, 0.40–0.59 fair, and <0.40 poor [21].

## 3. Results

From an initial sample of 321 subjects, 15 were excluded because they did not correctly complete the questionnaire, and so the final study sample consisted of 306 participants, of whom 193 (63.1%) were women and 113 (36.9%), men. The participants had an average age of 47 years, height of 168 cm, weight of 72 kg, and BMI of 25.4. The body stature values were always higher for the male participants (Table 1).

Similar patterns of results were obtained in each of the questionnaires used. For the FFI RSF Sp, the average score was 47.7 points for all participants; for the VASFA Sp questionnaire, it was 155 points; for the FAAMADL questionnaire, it was 51.5 points; and for the FAAMSPORT questionnaire, it was 14.5 points. Each participant required between 14 and 21 min to complete the FFI-RSF-Sp without direct assistance from the researchers. No concerns regarding comprehension or readability were reported.

According to the questionnaire results, individuals with some type of alteration presented higher dysfunction values compared to those with no such alteration (Table 2).

### 3.1. Structural Validity

**Figure 2:**
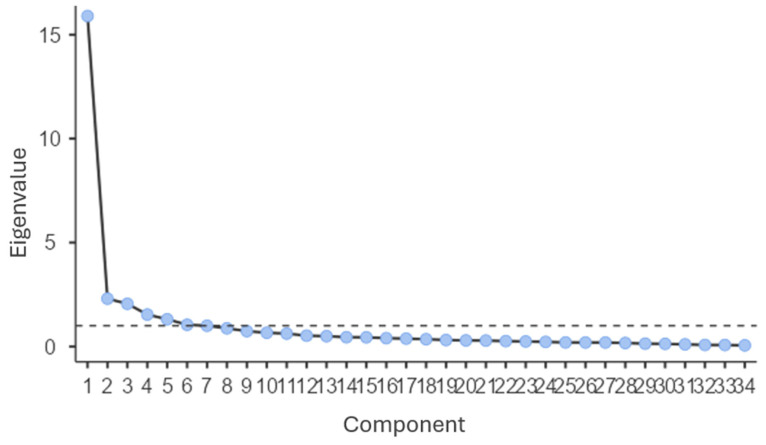
Scree plot showing eigenvalues by component.

Exploratory factor analysis confirmed the five factors comprising the questionnaire, as shown in the scree plot graph (Figure 2), with a factor solution that explained 67.6% of the total variance.

The model fit was assessed using confirmatory factor analysis, including all items from the original questionnaire with an RMSEA of 0.0785 and a TLI value of 0.874 (*p* < 0.001) [22].

### 3.2. Internal Consistency

The FFI-RSF-Sp presented excellent internal consistency, with a Cronbach’s alpha of 0.963 for the full instrument. The Cronbach’s alpha was 0.870 for the Pain subscale, 0.789 for Stiffness, 0.886 for Disability, 0.776 for Limitation, and 0.868 for Social aspects. All of these values were considered “good”.

### 3.3. Discriminatory Power

**Figure 3:**
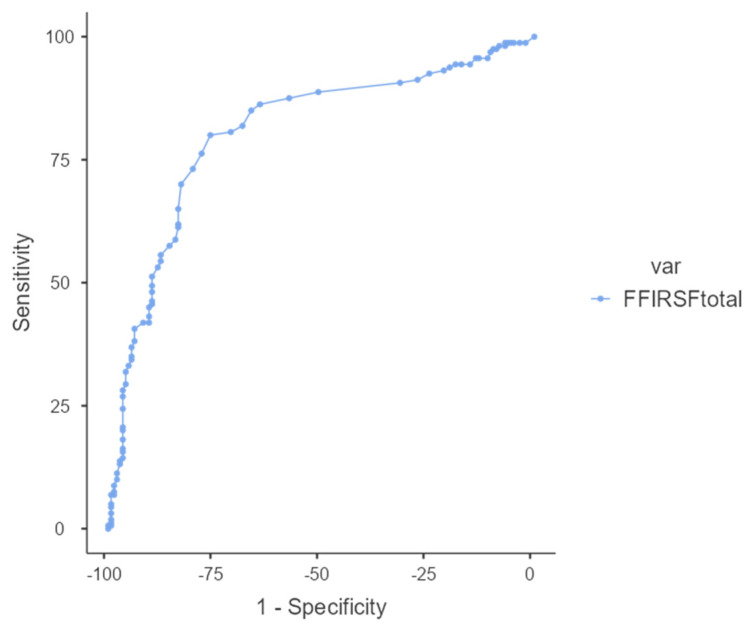
ROC curve showing the area under the curve of the Spanish FFI-RSF.

The area under the curve (AUC) obtained was 0.806 (*p* < 0.001), according to the ROC curve and as shown in Figure 3. The optimal cut-off value assumed for the Youden Index was 53.

### 3.4. Reliability

The FFI-RSF-Sp had good test–retest reliability, with a global ICC of 0.92 (95% CI; 0.62 to 0.97).

### 3.5. Criterion Validity

Good correlation values were obtained between the FFI-RSF-Sp and VASFA-Sp, with a Pearson’s coefficient of −0.651 (*p* < 0.001), and with the Spanish version of the FAAM ADL producing a Pearson’s coefficient of −0.737 (*p* < 0.001).

## 4. Discussion

The aim of this study was to develop a cross-cultural adaptation of the FFI RSF questionnaire into Spanish, thus generating a validated instrument for use by professionals and clinicians in daily practice. To this end, the present adaptation was compared with previously validated questionnaires of a similar scope and area of interest.

A noteworthy finding from our analysis of the completed questionnaires is that the female participants dedicate more than twice as much time as men (59 vs. 26 h per week) to physical activities, reflecting a significant difference between the sexes in their commitment to physical activity.

The results also indicate that those who use orthotic devices such as plantar supports reported lower FFI RSF scores, from which we conclude that the use of these devices improves foot function.

Another significant result is the difference in scores between healthy subjects and those who reported a health problem. In particular, those who reported foot pain and osteoarthritis presented the greatest difference in scores compared to healthy individuals. This can be interpreted as indicating a dysfunction, which is reflected in the three questionnaires administered, and therefore a decreased quality of life.

The FFI-RSF-Sp questionnaire presents certain advantages in the multidimensional assessment of foot function by incorporating the additional subscales of Stiffness and Quality of Life, which complement the traditional dimensions of Pain, Disability, and Functional Limitation. Our findings reveal that the FFI-RSF-Sp has robust measurement properties, and that it can be considered a promising resource for clinical practice and research in a Spanish-speaking population.

Exploratory factor analysis confirmed the questionnaire’s five-factor structure, consistent with the subscales of the original version: pain, stiffness, disability, functional limitation, and social or quality of life aspects, explaining 67.6% of the total variance. Confirmatory factor analysis yielded an RMSEA of 0.0785 and a TLI of 0.874, indicating acceptable model fit. These results are particularly relevant when compared with the cross-cultural adaptation of the original FFI into Spanish, which showed a poorer structural fit (comparative fit index of 0.78 and RMSEA of 0.23); this suggests that incorporating the new subscales into the RSF version may improve the representation of foot function.

In terms of internal consistency, the FFI-RSF-Sp presented an overall Cronbach’s alpha of 0.963, with subscale-specific values ranging from 0.776 to 0.886. These results demonstrate high item homogeneity and are comparable to those reported for the Norwegian adaptation of the FFI-RSF, which had a value of 0.97. Furthermore, a test–retest was conducted in a subsample of 20 participants with a 7-day interval, yielding an ICC of 0.92, which confirmed the instrument’s reliability, falling within the “excellent” range (ICC > 0.70). These results confirm that the FFI-RSF-Sp is a consistent and stable instrument over time, as is essential for use in longitudinal studies and in the monitoring of clinical interventions.

The criterion validity of the FFI-RSF-Sp was assessed by comparing its scores with those obtained from other questionnaires previously validated in Spanish, such as the VASFA-Sp [17] and the FAAM-ADL-Sp [16]. Moderate to strong correlations were found (r = −0.651 and r = −0.737, respectively, *p* < 0.001), supporting the ability of the FFI-RSF-Sp to adequately reflect foot function and the presence of dysfunction. These correlations are within a similar range to those observed in other cross-cultural adaptations of the FFI-RSF. The robustness of the correlations found in our study indicates that the FFI-RSF-Sp behaves comparably to other reference instruments, providing external validity and facilitating the comparison of results with different studies and in other cultural contexts.

The ROC curve assessment of discriminatory capacity revealed an area under the curve of 0.806 (*p* < 0.001), suggesting that the FFI-RSF-Sp effectively distinguishes between subjects with different levels of foot function. The optimal cutoff value observed enables us to distinguish subjects with significant alterations (scores > 53) from those with preserved function (scores < 53). This finding is fundamental for the clinical application of the questionnaire, since it provides a practical threshold for decision-making in patient management and follow-up. In comparison, other adaptations have reported discrimination values that fall within similar ranges, which corroborates the usefulness of the FFI-RSF-Sp in identifying dysfunction in daily practice.

The Spanish-language version of the FFI-RSF, with internal consistency and factor structure results similar to those observed in other cross-cultural adaptations, is thus a robust option for use in Spanish-speaking contexts. However, and as observed in other studies, a more precise evaluation of aspects such as measurement error and responsiveness requires additional research in specific clinical settings, considering populations with specific pathologies.

The strength of this study is the good validity and reliability results of the FFI-RSF questionnaire. This questionnaire, unlike the FAAM-ADL or VASFA, includes more domains, allowing clinicians and researchers to collect more relevant data from patients, not only recording quality of life or general foot-related function (FAAM-ADL and VASFA, respectively) but also pain and limitations. This makes the FFI-RSF useful for any type of patient or pathology.

Two of the limitations are the administration of the questionnaire in only two regions and the recruitment of the general population. Future studies should be carried out including more regions of the country and reliability studies in populations with specific pathologies and in longitudinal and patient follow-up studies. In this way, a more heterogeneous sample of individuals can be obtained, allowing for broader representation of the population. This approach also enables the possibility of designing the sampling process as a multicentre study. A potential course of action would be to establish collaborations with other centres interested in participating, in order to expand the sample and thereby increase its heterogeneity.

## 5. Conclusions

The data obtained in this study support the use of the FFI RSF-Sp questionnaire, showing it to be a high-quality, valid, and reliable instrument for assessing foot and ankle function. Its robust factor structure and high internal consistency, together with the strong correlations observed with other validated questionnaires, confirm its potential to improve clinical assessments.

## Figures and Tables

**Table 1:** Characteristics of participants.

	Total Sample (n = 306)	Men (n = 113)	Women (n = 193)
	Mean (SD)	Mean (SD)	Mean (SD)
Weight (kg)	71.8 (14.5)	81.5 (12.9)	66.1 (12.2)
Height (cm)	168 (9.75)	177 (7.62)	163 (6.75)
BMI	25.4 (4.17)	26.1 (3.85)	24.9 (4.29)
Age (years)	47.7 (17.8)	45.7 (18.3)	48.8 (17.4)
Sport (hours/week)	40.1 (2.92)	26 (2.92)	59 (2.62)
FFIRSF total	47.7 (22.6)	45.8 (19.4)	48.9 (24.3)
VASFA total	155 (47.3)	157 (44.8)	153 (48.8)
FAAMADL	51.5 (9.94)	52 (9.57)	51.2 (10.2)
FAAMSPORT	14.5 (4.1)	15.9 (4.1)	13.6 (3.9)

**Table 2:** Results of the self-administered questionnaires.

		FFI-RSF Mean (SD)	VASFA Mean (SD)	FAAM-ADL Mean (SD)
Diabetes	Yes	53.7 (25.2)	143 (47.3)	47 (13.1)
	No	47.1 (22.3)	156 (47.1)	52 (9.44)
Arthrosis	Yes	57.3 (27)	139 (39.9)	44.9 (10.9)
	No	45.3 (20.8)	158 (48.3)	53.1 (8.97)
Use of insoles	Yes	53.4 (24.8)	152 (41.2)	49.4 (10)
	No	42.3 18.9)	157 (52.5)	53.5 (9.49)
Foot pain	Yes	57.7 (22.5)	139 (39.4)	47.4 (10.3)
	No	36.8 (17.1)	171 (49.6)	56 (7.21)

## Data Availability

The data supporting the findings of this study are not publicly available due to privacy and ethical restrictions.
